# Additional nerve blocks are not superior to multiple-site infiltration analgesia in total knee arthroplasty under adductor canal block

**DOI:** 10.1186/s13018-021-02769-9

**Published:** 2021-10-13

**Authors:** Qianhao Li, Qinsheng Hu, Mohammed Alqwbani, Donghai Li, Zhouyuan Yang, Qiuru Wang, Pengde Kang

**Affiliations:** Department of Orthopaedics Surgery, West China Hospital, Sichuan University, 37# Guoxue Road, Chengdu, 610041 Sichuan People’s Republic of China

**Keywords:** Total knee arthroplasty, Adductor canal block, Multiple-site infiltration analgesia, Additional nerve blocks, Analgesia, Cost-effectiveness

## Abstract

**Background:**

Adductor canal block (ACB) with additional nerve blocks (ANBs) is reported to provide adequate analgesia and enhanced functional rehabilitation in total knee arthroplasty (TKA). The present study aims to evaluate whether ANBs are superior to multiple-site infiltration analgesia (MIA) in patients undergoing TKA under ACB.

**Methods:**

We enrolled 530 patients undergoing primary TKA from 2015 to 2019 at our institution in this retrospective cohort study. Patients were divided into two groups: Group A was treated with ANBs + ACB; Group B was treated with MIA + ACB. Primary outcomes were pain scores and morphine consumption. Functional recovery was the secondary outcome. Other outcomes included satisfaction score, cost-effectiveness, adverse events, and length of hospital stay (LOS).

**Results:**

Pain scores at rest and morphine consumption were slightly lower in the ANBs + ACB group than in the MIA + ACB group. No significant difference was found in functional recovery, post-operative complications or LOS between the groups. Meanwhile, the cost of analgesic intervention in the MIA + ACB group was less than that in the ANBs + ACB group.

**Conclusion:**

The present study suggests that ANBs do not provide superior pain relief compared to MIA for patients undergoing TKA under ACB.

**Trial registration:**

Chinese Clinical Trial Registry, ChiCTR2100043227. Registered 9 February 2021, https://www.chictr.org.cn/showproj.aspx?proj=121745.

## Introduction

Total knee arthroplasty (TKA) is currently the most effective treatment for alleviating persistent pain in patients with end-stage knee arthritis [1]. However, almost 57% of patients complain about varying degrees of post-operative pain [2, 3]. Mobilization and rehabilitation can be retarded if the pain is poorly controlled, which may also induce adverse events, such as venous thromboembolism (VTE), pneumonia, dyssomnia, anxiety and readmission [4]. Perioperative analgesia should be applied to enhance the recovery and achieve the expected satisfaction of patients after surgery.

The American Society of Anesthesiologists (ASA) recommends multimodal analgesia strategy for acute pain in place of the extensive use of opioids, which has been connected with complications such as nausea, vomiting, sedation and respiratory depression [5]. Multimodal pain management includes oral analgesics, patient-controlled analgesia (PCA), peripheral nerve block (PNB) and multiple-site infiltration analgesia (MIA). Among these pain management methods, adductor canal block (ACB), which is regarded as a promising alternative to femoral nerve block (FNB), demonstrates gains in quadricep muscle strength and pain relief [6, 7]. The ACB blocks the vastus medialis and saphenous nerves, dominating the anterior and medial aspect of the knee joint. For the sake of satisfactory analgesia, ACB is generally associated with other techniques to remedy the limitation of nerves not targeted in the posterior and lateral sides [8, 9].

MIA, which is widely accepted, is achieved by injecting a mixture of local anaesthetics, epinephrine, corticosteroids, anti-inflammatory drugs and opioids into the articular cavity [10, 11]. When MIA is coadministered with ACB, it shows advantages over MIA alone in terms of the analgesic efficacy [8, 12]. Similarly, adding additional nerve blocks (ANBs) to ACB improves early outcomes after TKA [9, 13, 14]. For instance, infiltration of the interspace between the popliteal artery and capsule of the knee (iPACK) innervates the posterior capsule of the joint, indicating perfect integration with the ACB [14, 15]. The lateral femoral cutaneous nerve (LFCN) and the obturator nerve (ON) thread the knee and are considered adequate targets for nerve block to complement the ACB [16, 17]. By means of experimenting with divergent combinations of ACB and ANBs, these analgesic methods result in substantially better surgical outcomes, despite the higher cost that may exist in clinical practice [18].

Since little is known about whether ANBs + ACB is superior to MIA + ACB, this study aims to determine whether ANBs + ACB is superior to MIA + ACB by comparing patients’ post-operative pain, functional rehabilitation, cost-effectiveness and complications during TKA for the two approaches.

## Materials and methods

This study was designed as a retrospective cohort study. The protocol was approved by the institutional review board of the authors’ affiliated institutions. Informed consent was obtained from each patient.

### Patients

From January 2015 to November 2019, patients in our department who were undergoing primary unilateral TKA were eligible for recruitment. They were adults older than 18 years with a body mass index (BMI) of 18–35 kg/m2 and an American Society of Anesthesiologists physical status classification of I–III.

The exclusion criteria were: (1) knee flexion deformity ≥ 30°, varus–valgus deformity ≥ 30°; (2) allergy to morphine, local anaesthetics or other drugs used in this study; (3) chronic opioid consumption for more than 3 months prior to surgery; (4) diagnosis of traumatic arthritis, rheumatoid arthritis, septic arthritis or prior knee surgery; (5) mental illness or cognitive impairment making communication or comprehension difficult; and (6) thrombolytic events (deep vein thrombosis, pulmonary embolus, myocardial infarction and cerebrovascular accident).

### Group allocations

Patients were admitted by one medical team in our institution, and we divided them into 2 groups. The only difference between the groups was the nerve block approach. The ANBs + ACB group was treated with ACB combined with ANBs (iPACK/LFCNB/ONB + LFCNB/iPACK + LFCNB), and the other group was treated with ACB and MIA.

### Analgesic management and surgical protocol

All patients’ basic characteristics were recorded preoperatively: age, sex, BMI, and surgery side, pain scores, quadricep strength, ASA status, and range of motion and Knee Society Score (KSS). Loxoprofen (60 mg, b.i.d.) was taken as pre-emptive analgesia.

One senior surgeon performed the TKA procedures after general anaesthesia was applied. A medial parapatellar approach was adopted by making an anterior midline skin incision. The surgeon inserted prostheses (DePuy PFC and Stryker Triathlon) with cementing techniques. Patients were given 1 g of tranexamic acid intravenously during the surgery.

An experienced anaesthetist finished nerve blocks via a high-frequency linear array ultrasonic transducer, injecting an anaesthetic cocktail (0.2% ropivacaine and 2.0 μg/ml epinephrine) into the nerve region. ACB and additional nerve blocks were performed by single-shot injections preoperatively, while MIA was implemented by the surgeon during the surgery.

ACB: After scanning the middle of the thigh to locate the adductor canal, the anaesthetist embedded a 22-gauge, 100-mm needle in a lateral-to-medial plane. Three millilitres of isotonic saline was injected to ensure the target position of the saphenous nerve and the nerve to the vastus medialis. Then, 20 ml of the anaesthetic cocktail was injected.

iPACK: Under ultrasound guidance, the popliteal artery and the capsule of the knee were identified by using an in-plane approach to the intercondylar fossa. Twenty millilitres of the anaesthetic cocktail could be injected into the space provided the needle was in the correct place, which was confirmed by 3 ml of isotonic saline.

LFCNB: The ultrasonic transducer helped detect the lateral femoral cutaneous nerve along the inguinal crease deep in the fascia lata. Then, the anaesthetist inserted 10 ml of medication around the nerve.

ONB: A 22-gauge, 100-mm needle oriented the obturator nerve after finding the fascial planes of the pectineus and adductor muscles through ultrasonic guidance. Five millilitres of the cocktail was injected between the pectineus and adductor brevis muscles, and another 5 ml was injected between the adductor brevis and adductor magnus muscles.

MIA: Patients were given the same cocktail (0.2% ropivacaine and 2.0 μg/ml epinephrine, total 60 ml) periarticularly via a multisite technique during the operation. Twenty millilitres of cocktail for each infiltration site was injected into the posterior aspect of the capsule (prior to placement of the prosthesis), medial and lateral collateral ligaments (prior to component implantation) and quadriceps and retinacular tissues (after implantation).

All patients received ice compression but no analgesia pump when they returned to the ward. Loxoprofen (60 mg, b.i.d.) and prolonged-release oxycodone hydrochloride tablets (10 mg, b.i.d.) were prescribed to manage post-operative pain. Morphine hydrochloride (10 mg) was injected subcutaneously only if patients were unable to bear pain. To avoid venous thromboembolism (VTE), every patient was administered enoxaparin (0.2 ml) 12 h after surgery and then 0.4 ml every 24 h afterwards until discharge. After leaving the hospital, rivaroxaban (10 mg, qd) was taken orally for two weeks to continue VTE prophylaxis. Patients received lower extremity strength training when they were capable of moving their lower limbs. Before discharge, patients were expected to walk with walking aids.

### Outcomes

Primary outcomes were pain scores measured by the visual analogue scale (VAS, 0 = no pain, 10 = worst pain) [19] and morphine hydrochloride use before discharge. Anterior and posterior knee pain at rest and with activity (knee flexion of 45°) were evaluated on the first and second days after surgery.

The secondary outcome was functional recovery of the knee, including range of motion, quadricep strength, daily ambulatory distance and KSS function score during hospitalization and follow-up. Moreover, the length of hospital stay was defined as the time from the end of surgery to discharge, and a patient’s satisfaction score (assessed by VAS, 0 = least satisfied, 100 = most satisfied) was also recorded at 48 h post-operatively. In our hospital, the cost of equipment and supply to provide nerve block is 195 CNY each position. Ropivacaine (100 mg) costs 45.78 CNY and epinephrine (1 mg) costs 4.8 CNY. Any occurrences of complications composed of nausea, vomiting, falls, infection, calf paraesthesia, venous thrombosis, or cerebrovascular and cardiovascular events or wound adverse events (ooze, swelling, delayed healing) were documented in detail. Patient mortality and readmission rate were counted at the 6-month follow-up.

### Statistical analysis

Statistical data were calculated using SPSS 25.0 (IBM Corp., Armonk, NY, USA). Quantitative variables are represented as the mean ± standard deviation (SD), and qualitative variables are represented as the number and percentage (%). Differences in continuous data between groups were analysed by Student’s t-test or Mann–Whitney U test, after confirming their distribution types (normal or non-normal distribution). Chi-squared and Fisher’s exact tests were used to analyse categorical data. Differences of *p* < 0.05 were considered significant.

## Results

A total of 530 patients undergoing primary unilateral TKA were divided into two groups depending on analgesic strategies. Of these, 290 patients were treated with ACB and ANBs, while the other 240 patients were assigned to the ACB and MIA group. With regard to the patients who were treated with ANBs + ACB, various additional nerve block proposals were made, and 110 patients received ACB + iPACK, 80 received ACB + LFCNB, 50 received ACB + ONB + LFCNB and 50 received ACB + iPACK + LFCNB.

As shown in Table 1, there were no significant differences between the ANBs + ACB and MIA + ACB groups in preoperative baseline characteristics or the duration of surgery. The VAS scores for the ANBs + ACB and MIA + ACB groups at rest were (POD1: 3.2 vs. 3.4, *p* = 0.09; POD2: 2.8 vs. 3.0, *p* = 0.18; at discharge: 2.0 vs. 2.1, *p* = 0.13), respectively. Morphine consumption during hospitalization between the groups was slightly lower in the ANBs + ACB group (POD1: 9.6 vs. 10.5, *p* = 0.15; POD2: 2.1 vs. 2.9, *p* = 0.08; POD3: 0.2 vs. 0.3, *p* = 0.51; total use: 11.9 vs. 13.2, *p* = 0.13), although there was no significant difference. Patients in the ANBs + ACB and MIA + ACB groups had similar VAS scores with activity (POD1: 4.3 vs. 4.4, *p* = 0.50; POD2: 3.5 vs. 3.6, *p* = 0.38; at discharge: 2.5 vs. 2.4, *p* = 0.23, respectively) (Table 2).

**Table 1:** Patient clinical and demographic characteristics

Characteristics	ANBs + ACB	MIA + ACB	*p-*value
Age (years)	64.2 ± 8.4	63.5 ± 7.7	0.32
Gender (M/F)	82/208	67/173	0.93
Body mass index (kg/m^2^)	25.4 ± 5.6	24.3 ± 7.7	0.06
Surgery side (right/left)	154/136	116/124	0.27
Analgesia method (cases, n)	290	240	–
ACB + iPACK	110	–	
ACB + LFCNB	80	–	
ACB + ONB + LFCNB	50	–	
ACB + iPACK + LFCNB	50	–	
ACB + MIA	–	240	
Pre-VAS pain scores	4.5 ± 1.8	4.7 ± 1.6	0.18
Pre-knee ROM	92.4 ± 10.3	94.0 ± 11.3	0.09
Pre-KSS function score	25.2 ± 8.4	24.6 ± 9.6	0.44
Pre-quadricep strength	4.9 ± 0.8	4.8 ± 0.7	0.13
ASA status (I/II/III)	48/146/96	32/128/80	0.57
Duration of surgery (min)	81.2 ± 16.5	82.9 ± 15.4	0.22

*ANBs* additional nerve blocks; *ACB* adductor canal block; *MIA* multiple-site infiltration analgesia; *iPACK* infiltration between the popliteal artery and the capsule of the posterior knee; *LFCNB* lateral femoral cutaneous nerve block; *ONB* obturator nerve block; *VAS* visual analogue score; *ROM* range of motion; *ASA* American Association of Anesthesiologists; *KSS* Knee Society Score

**Table 2:** Pain scores and analgesic drugs consumption

Parameters	ANBs + ACB	MIA + ACB	*p*-value
Pain VAS scores At rest			
POD1	3.2 ± 1.2	3.4 ± 1.5	0.09
POD2	2.8 ± 1.9	3.0 ± 1.4	0.18
At discharge	2.0 ± 0.7	2.1 ± 0.8	0.13
With activity			
POD1	4.3 ± 1.8	4.4 ± 1.6	0.50
POD2	3.5 ± 1.2	3.6 ± 1.4	0.38
At discharge	2.5 ± 1.0	2.4 ± 0.9	0.23
Morphine (mg)			
POD1	9.6 ± 7.0	10. 5 ± 7.2	0.15
POD2	2.1 ± 4.4	2.9 ± 6.0	0.08
POD3	0.2 ± 1.6	0.3 ± 1.9	0.51

*ANBs* additional nerve blocks; *ACB* adductor canal block; *MIA* multiple-site infiltration analgesia; *VAS* visual analogue score; *POD1* post-operative day one; *POD2* post-operative day two; *POD3* post-operative day three

All the patients completed early rehabilitation exercise routines and were assessed for functional recovery in hospital and at follow-up. Knee ROM, quadricep strength and daily ambulatory distance were comparable between the two groups (*p* > 0.05). Patients in the ANBs + ACB group stayed in the hospital for 66.4 ± 8.6 h post-operatively, and those in the MIA + ACB group stayed for 65.5 ± 7.5 h (*p* > 0.05). There was no significant difference in the KSS or patient satisfaction scores between the two groups (*p* > 0.05). However, the average cost of analgesic intervention in the MIA + ACB group was significantly lower than that in the ANBs + ACB group (291.36 vs. 508.05, *p* < 0.01) (Table 3).

**Table 3:** Post-operative knee functional recovery and expenditure

Parameters	ANBs + ACB	MIA + ACB	*p*-value
Knee ROM (degrees)			
POD1	80.2 ± 15.1	81.1 ± 16.3	0.51
POD2	90.4 ± 14.9	88.9 ± 13.6	0.23
At discharge	101.2 ± 13.2	100.5 ± 11.6	0.52
Po-6 month	116.6 ± 9.3	116.2 ± 10.2	0.64
Quadricep strength			
POD1	3.5 ± 0.9	3.6 ± 0.8	0.18
POD2	3.9 ± 0.8	4.0 ± 0.9	0.18
At discharge	4.5 ± 0.6	4.5 ± 0.5	1.00
Po-6 month	4.9 ± 0.6	4.9 ± 0.7	1.00
Daily mobilization distance (m)			
POD1	9.0 ± 8.9	8.7 ± 8.8	0.70
POD2	17.1 ± 12.5	16.4 ± 13.4	0.53
At discharge	30.5 ± 13.4	31.2 ± 14.2	0.56
Po-6 month	864 ± 132	882 ± 124	0.11
Post-operative hospital stay (h)	66.4 ± 8.6	65.5 ± 7.5	0.20
Patient satisfaction scores	90.3 ± 9.3	91.4 ± 8.5	0.16
KSS function score			
At discharge	33.5 ± 6.3	34.7 ± 5.2	0.12
Po-6 month	57.4 ± 5.6	56.7 ± 5.8	0.16
Cost of analgesic intervention (CNY)	508.05 ± 92.9	291.36 ± 0	< 0.01

*ANBs* additional nerve blocks; *ACB* adductor canal block; *MIA* multiple-site infiltration analgesia; *VAS* visual analogue score; *ROM* range of motion; *POD1* post-operative day one; *POD2* post-operative day two; *KSS* Knee Society Score

Concerning complications, the number of patients with PONV was similar in the two groups (*p* > 0.05), and they were treated with metoclopramide dihydrochloride injections to alleviate the symptoms. Differences in the incidence of falls after surgery, wound complications and any venous thrombotic events were not significant (*p* > 0.05). Four patients in the ANBs + ACB group and 1 patient in the MIA + ACB group experienced slight calf paraesthesia (*p* > 0.05). In addition, no other adverse events were found, such as infection, readmission, cerebrovascular events, cardiovascular events or 6-month mortality (Table 4).

**Table 4:** Post-operative complications (n, %)

Adverse events	ANBs + ACB	MIA + ACB	*p*-value
PONV	98 (33.8)	84 (35.0)	0.77
Wound complications	15 (5.2)	14 (5.8)	0.74
Falls after surgery	8 (2.8)	3 (1.3)	0.36
Calf paraesthesia	4 (1.4)	1 (0.4)	0.49
Calf muscular venous thrombosis	18 (6.2)	16 (6.6)	0.83
Venous thrombotic events	0 (0)	1 (0.4)	1.00
Infection	0 (0)	0 (0)	–
Cerebrovascular events	0 (0)	0 (0)	–
Cardiovascular events	0 (0)	0 (0)	–
6-month mortality	0 (0)	0 (0)	–
6-month readmission	0 (0)	0 (0)	–

*ANBs* additional nerve blocks; *ACB* adductor canal block; *MIA* multiple-site infiltration analgesia; *PONV* post-operative nausea and vomiting

## Discussion

This retrospective study shows that ANBs are not superior to MIA in total knee arthroplasty under ACB. We found no difference between the two groups in pain scores, morphine consumption, functional rehabilitation or adverse events after conducting these regional anaesthetic techniques. The combination of MIA and ACB is less expensive than ANBs + ACB group, making this surgical analgesic technique more cost-effective.

TKA is a major orthopaedic surgery, and acute pain and complications occasionally occur after surgery [1, 3]. In this context, adequate pain management plays an important role in improving patient satisfaction and the quality of early recovery [20]. Several methods have been attempted to obtain a combination of multimodal analgesia [4, 21]. PNB and MIA are efficient strategies in conjunction with oral analgesics, and they are routine choices widely applied in practice [20]. MIA, which is more commonly use during TKA, requires surgeons to inject an anaesthetic cocktail into the joint from deep to superficial layers and achieves satisfactory outcomes for analgesia [12, 22]. Given the purpose of comprehensive treatment, MIA with ACB is recommended for reasons of better pain relief and less opioid consumption [12, 23].

FNB is one of the standard methods of PNB, considerably reducing pain compared with PCA [24]. Even so, motor blockade and quadriceps insufficiency exist, which prolong the period of functional recovery after TKA [25]. ACB has gained in popularity thanks to its analgesia properties commensurate with FNB, while preserving muscle strength and mitigating the risk of falls [6, 7]. To improve the efficacy of a single ACB shot, ANBs could be added as an alternative to the mix of MIA and ACB. LFCNB and iPACK offer adjuvant nerve blocks for ACB, which also show optimal early rehabilitation without severe adverse events [9, 13–16]. There are a number of trials identifying MIA + ACB outcomes (Table 5), but ANBs + ACB is rarely referred as a comparison [8, 12, 14, 15, 23, 26–29]. Therefore, we designed this study.

**Table 5:** Review of related papers on literature

Author	Year	Techniques (group I vs II)	Outcomes
Nader [23]	2016	MIA vs ACB + MIA	Group II reduces pain and opioids
Gwam [26]	2017	MIA vs ACB + MIA	Comparable outcomes
Goytizolo [12]	2019	MIA vs ACB + MIA	Group II shows lower pain score
Sawhney [8]	2016	ACB vs ACB + MIA	Group II shows lower pain score
Sankineani [27]	2018	ACB vs ACB + MIA	Group II shows lower pain score
Chuan [28]	2019	FTB + MIA vs ACB + MIA	Comparable outcomes
Shah [29]	2014	FNB + MIA vs ACB + MIA	Group II shows better ambulation
Kertkiatkachorn [14]	2020	ACB + iPACK vs ACB + MIA	Group II needs less opioids
Jung [15]	2020	ACB + iPACK vs ACB + MIA	Group II shows higher pain score

*MIA* multiple-site infiltration analgesia; *ACB* adductor canal block; *FTB* femoral triangle block; *FNB* femoral nerve block; *iPACK* infiltration between the popliteal artery and the capsule of the posterior knee

We selected three types of additional nerve blocks (LFCNB, iPACK and ONB) we had used in TKA; some of these nerve blocks have been introduced in previous studies [9, 14, 17]. We finally included iPACK, LFCNB, ONB + LFCNB and iPACK + LFCNB as ANBs. In this study, the ANBs + ACB group showed no superiority in pain relief compared with the MIA + ACB group. There was no significant difference in VAS score or morphine use before discharge, although the two groups both achieved the goal of valid analgesia. Post-operative pain, if controlled, can contribute to early functional recovery and fewer complications, such as venous thrombosis, myocardial infarction and pneumonia [4]. Hence, the relatively low pain scores between the two groups may allow patients to try functional exercises earlier and increase their levels of satisfaction. All the indicators of rehabilitation in the ANBs + ACB group obtained similar results to those in the MIA + ACB group after mobilization. Meanwhile, the incidence of falls showed no significant difference, suggesting that our combinations of multiple nerve blocks would not influence the muscle strength of the lower limb.

The cost-effectiveness of analgesic interventions was taken into consideration. Corman et al. [18] reported a higher cost of PNB than periarticular infiltration. In our medical centre, we also estimated the materials of nerve blocks and local infiltration, including equipment, supplies and medications. The average cost of MIA + ACB (291.36 CNY) was lower than the cost of ANBs + ACB (508.05 CNY). Thus, using the MIA + ACB technique may be cost-effective in TKA without sacrificing efficacy and safety. In addition, several studies have reported that PNB may be correlated with neurologic compromise and heel ulcers [20, 25, 30], while MIA also has the risk of systemic toxicity or cardiac arrhythmia due to the invasive procedure. In contrast to these previous findings, no such severe complications occurred in our study. A few patients experienced calf paraesthesia, which resolved spontaneously within weeks.

Our study has some limitations. First, the study was retrospective which meant that we collected data reliant on our medical records. Thus, we may have overlooked the existence of some undocumented outcome indicators, such as adverse events. PNB-related complications could require longer follow-up to determine. Although this was the first study to assess the efficacy of ACB when combined with ANBs or MIA in TKA to our knowledge, in terms of study design, a prospective study with a standard protocol should be created to increase evidence. Second, there was a lack of measurement of systemic toxicity, such as serum levels, although we did not observe serious local anaesthetic intoxication. Finally, we only counted the difference between ANBs + ACB and MIA + ACB, and the subfractions of ANBs were not considered.

## Conclusions

ANBs + ACB and MIA + ACB are both effective approaches to alleviate pain and speed functional recovery after TKA. However, ACB with ANBs is not superior to the MIA + ACB approach in alleviating pain or speeding recovery; further, ANBs + ACB is more costly and technically difficult. Additional studies should explore better ways to manage pain and maximize functional recovery for patients.

## Data Availability

The datasets used and/or analysed during the current study are available from the corresponding author on reasonable request.

## Abbreviations

ACB: Adductor canal block
ANBs: Additional nerve blocks
TKA: Total knee arthroplasty
MIA: Multiple-site infiltration analgesia
LOS: Length of hospital stay
VTE: Venous thromboembolism
ASA: The American Society of Anesthesiologists
PCA: Patient-controlled analgesia
PNB: Peripheral nerve block
FNB: Femoral nerve block
iPACK: The infiltration of the interspace between the popliteal artery and capsule of the knee
LFCNB: Lateral femoral cutaneous nerve block
ONB: Obturator nerve block
BMI: Body mass index
KSS: Knee Society Score
VAS: Visual analogue scale
POD: Post-operative day
ROM: Range of motion
PONV: Post-operative nausea and vomiting
